# Cluster of Speaking-Up Behavior in Clinical Nurses and Its Association With Nursing Organizational Culture, Teamwork, and Working Condition: A Cross-Sectional Study

**DOI:** 10.1155/jonm/9109428

**Published:** 2024-11-15

**Authors:** Eunhee Lee, Hyunjeong Kwon

**Affiliations:** ^1^College of Nursing, Sungshin Women's University, Seoul, Republic of Korea; ^2^College of Nursing, Keimyung University, Daegu, Republic of Korea

**Keywords:** safe culture, teamwork, voice behavior, withholding, working condition

## Abstract

**Introduction:** Nurses, as frontline healthcare professionals, play a crucial role in ensuring patient safety, making their ability to speak up imperative. However, there are limited studies categorizing nurses based on their speaking-up behaviors and comparing their organizational characteristics. This study aimed to identify patterns of nurses' speaking-up behaviors and examine differences in organizational cultures, teamwork climates, and working conditions according to these patterns.

**Methods:** A cross-sectional survey was conducted, involving 597 nurses directly participating in nursing care in Korean hospitals. The Speaking Up about Patient Safety Questionnaire (SUPS-Q), Nursing Organizational Culture Questionnaire, and Safety Attitude Questionnaire-Korean version (SAQ-K) were employed to measure nurses' speaking-up-related behaviors, organizational culture, teamwork climate, and working conditions. Cluster analysis was used to identify clusters of nurses based on their speaking-up-related behavior. Differences in nursing organizational culture, teamwork climate, and working conditions among clusters were analyzed using one-way analyses of variance.

**Results:** Three clusters of nurses were identified based on their speaking-up-related behaviors. Cluster 1 (35%) showed high perceived concerns, moderate withholding, and speaking up, while Cluster 2 (37%) had moderate concerns, low withholding, and high speaking up. Cluster 3 (28%) had moderate concerns but low withholding and speaking up. Clinical experience significantly differed among clusters, with less experienced nurses predominantly in Cluster 1. Cluster 2 had the most collaborative culture, best teamwork climate, and working conditions.

**Conclusion:** Ensuring patient safety requires nurses to speak up about patient safety concerns. Creating safe working environments and fostering an organizational culture that prioritizes patient safety are essential steps in promoting nurses' willingness to speak up about patient safety.

## 1. Introduction

Nurses, as frontline healthcare professionals, play a pivotal role in performing direct patient care, advocating for patient needs and collaborating with interdisciplinary teams. Since they are positioned to observe early signs of unsafe conditions in healthcare delivery, their ability to communicate promptly and effectively is critical for patient safety [1, 2]. Several studies have highlighted that assertive and insistent sharing of information regarding patient health and safety concerns, known as speaking up, has an immediate preventive effect on adverse events such as medical errors and infections [3–5]. Despite the crucial role of nurses' effective communication in ensuring patient safety, nurses often face challenges in speaking up about patient safety. Sometimes, they remain silent even though patient safety is jeopardized [3, 6, 7].

The speaking-up behavior of nurses can be affected by various factors, ranging from individual traits (such as competency and previous experience) to organizational factors (such as interpersonal dynamics and cultures) [5, 8–10]. Many previous studies have emphasized the impact of external factors such as teamwork, organizational structure or culture, and work environment on nurses' speaking up rather than internal factors such as individual perceived effectiveness and confidence [11, 12]. Among several organizational factors, a hierarchical structure has been revealed as a critical factor for inhibiting nurses' speaking up [5, 10]. In a hierarchical structure, voicing concerns to superiors within the same profession can be challenging, and voicing concerns about patient safety across professional boundaries can be even more difficult [13–17]. A hierarchical structure immediately hinders nurses' speaking up and simultaneously accelerates nurses' withholding voices, lowering their psychological safety [4, 18]. In addition to a hierarchical structure, poor work environments with high workloads and resource constraints have been identified as barriers to raising patient concerns by nurses [19, 20]. An overwhelming workload could lead nurses to remain silent to avoid the additional work to address threats to patient safety [21].

Although nurses' speaking up is hindered by organizational factors, it can be facilitated by several organizational factors, such as teamwork and relationships between team members, and support organizational culture [11, 12]. Teamwork is critical to providing safe and effective healthcare care and has been reported to influence speaking up [12, 22]. Since most clinical situations requiring voice behavior occur within healthcare teams, positive teamwork and relationships between team members can increase nurses' psychological safety, thus promoting speaking up [4, 22, 23]. In addition to its direct relationships with voice targets, organizational culture or climate has been associated with nurses' speaking up [6, 10, 22]. While hierarchical organizational culture inhibits nurses' speaking up, a safety culture that is nonjudgmental, nonpunitive, and supportive of open communication with a respectful organizational culture enables nurses to speak up about patient safety [24, 25].

The decision to speak up or not is dynamic and situation-specific [13, 15]. Nurses' speaking up about patient safety is particularly linked to their perception of situations they encounter because they are positioned to primarily observe situations that cause harm to patients. The more the risk of a situation is perceived, the more likely the healthcare professionals are to speak up about patient safety [13]. In addition to speaking up, perception of situations is also closely linked to withholding voice as well [7, 15]. Withholding voice, referring to an intentional behavior not to verbalize ideas, information, and opinions for improving patient safety, is a more serious behavior than simply not speaking up. Withholding a voice is not equivalent to not speaking up; however, withholding a voice may be negatively associated with speaking up [7, 9]. This suggests that perceptions of situations, withholding voice, and speaking up are closely connected. To fully understand nurses' speaking-up behavior, it is important to consider these interrelated aspects comprehensively rather than examining each concept independently.

However, existing research has primarily focused on identifying factors influencing speaking up or withholding voices, without exploring the relationship between situational perception, speaking up, and withholding. This approach has limited the comprehensive understanding of differences in nurses' behaviors based on their specific behavioral or perceptual patterns. To address this gap, it is essential to classify behavioral clusters related to speaking up by examining the interconnected aspects of speaking up, withholding, and perception. Furthermore, it is necessary to explore whether organizational factors, such as culture, teamwork, and working conditions, appear differently across these behavioral clusters. Thus, our study addresses two main questions.

1. What are the distinct clusters of nurses based on their speaking-up-related behaviors?
2. How do organizational culture, teamwork climate, and working conditions differ across these clusters?

The aim of this study was to classify distinct clusters of nurses based on their speaking up-related behaviors and to examine differences in organizational culture, teamwork climate, and working conditions across these clusters. Through this, we expect to provide valuable insights into establishing tailored strategies aimed at enhancing nurses' speaking-up behaviors according to clusters.

## 2. Materials and Methods

### 2.1. Study Design

This cross-sectional survey was conducted in accordance with the Strengthening the Reporting of Observational Studies in Epidemiology guidelines.

### 2.2. Participants

This study included 597 nurses who provided direct care for patients in hospitals. Cluster analysis requires a sample size at least 70 times the number of variables to ensure accuracy [26]. As we used three variables for clustering, a minimum of 210 participants was needed. This study recruited more participants than the minimum requirement to account for the diversity of work environments. Since we investigated speaking-up behaviors about patient safety concerns, this study only included nurses directly caring for patients, excluding nurses working in departments not directly related to patient care, such as those working in the administrative department. This study included nurses who worked in various departments, not only in general care units (such as the general ward and the integrated nursing unit) but also in special care units such as the intensive care unit (ICU), emergency room (ER), and operating room (OR).

### 2.3. Data Collection

Data collection was initiated by posting a recruitment announcement on a nursing website, inviting nurses who met the inclusion criteria to participate voluntarily. Nurses who agreed to participate were provided with access to the online survey. An online survey was conducted to facilitate broad participation from nurses working in diverse healthcare settings across various regions. The survey was available for one month in March 2024, allowing participants to complete it conveniently.

### 2.4. Variables

General characteristics included gender, age, clinical experience, hospital type, hospital location, working department, and experience of speaking-up education. In South Korea, nurses with three or more years of experience are classified as experienced nurses by the government [27], a classification that we also adopted to distinguish between novice and experienced nurses. Hospital types were classified into tertiary hospitals, general hospitals, and hospitals in South Korea, based on bed size and hospital characteristics. This study included nurses from various work departments, special care units, and general care units. The integrated unit is a type of hospitalization unit with more than twice the number of nurse staffing compared with the general ward. It has been implemented in South Korea since 2015 to reduce the caregiving burden and improve the quality of care [28].

#### 2.4.1. Speaking-Up-Related Behavior

Speaking-up-related behavior was measured using the Speaking Up about Patient Safety Questionnaire (SUPS-Q) developed by Richard, Pfeiffer, and Schwappach [29]. Speaking-up-related behavior consisted of 11 items of three subdomains: (1) frequency of perceived safety concerns (3 items), (2) frequency of withholding voice by not speaking up in specific situations (4 items), and (3) frequency of speaking up (4 items). Responses for speaking-up-related behavior were measured on a 5-point response scale based on the frequency of the behavior over the last four weeks: 1 = never (0 times), 2 = rarely (1-2 times), 3 = sometimes (3–5 times), 4 = often (6–10 times), and 5 = very often (more than 10 times). The score for each subdomain ranged from 1 to 5. During the development of the SUPS-Q tool, the Cronbach's *α* value was reported as 0.73 for perceived concerns, 0.76 for withholding voice, and 0.85 for speaking up [29]. In this study, Cronbach's *α* values were 0.80, 0.86, and 0.87, respectively.

#### 2.4.2. Nursing Organizational Culture

Nursing organizational culture was assessed using a 20-item instrument developed by Kim, Han, and Kim [30], based on the Competing Value Framework proposed by Cameron and Quinn [31]. This framework categorizes organizational culture into four main quadrants: (1) clan culture, which emphasizes collaboration, cohesion, and involvement; (2) adhocracy culture, characterized by innovation and creativity; (3) market culture, which focuses on competition and productivity; and (4) hierarchical culture, which prioritizes structure, control, and formal processes. These quadrants represent tensions between internal and external factors, stability and control, and flexibility and change. Cronbach's *α* value in Kim, Han, and Kim [30] was 0.88 overall, 0.88 for collaborative culture, 0.83 for creative culture, 0.78 for hierarchical culture, and 0.72 for competitive culture. Cronbach's *α* value in this study was 0.85 overall, 0.89 for collaborative culture, 0.83 for creative culture, 0.73 for hierarchical culture, and 0.50 for competitive culture.

#### 2.4.3. Teamwork Climate and Working Conditions

Teamwork climate and working conditions were measured using the Safety Attitude Questionnaire-Korean version (SAQ-K) developed by Jeong et al. [32]. Teamwork climate refers to the quality of collaboration among team personnel, assessed using five items [32]. Working conditions, related to the quality of the work environment and logistical support, were measured using four items addressing sufficient staffing, training systems, information accessibility, and adequate supervision [32]. Both variables were measured on a 5-point Likert scale ranging from 1 (strongly disagree) to 5 (strongly agree). The Cronbach's *α* values for teamwork climate and working conditions were 0.84 and 0.76, respectively, during tool development [32]. This study's Cronbach's *α* value was 0.86 for teamwork climate and 0.81 for working conditions.

### 2.5. Ethical Consideration

This study was approved by Hallym University in April 2023 (no. HIRB-2023-015). Data collection commenced after receiving ethics approval and was completed in March 2024. Written informed consent was obtained from all participants prior to their participation in the survey. In addition, the primary investigator obtained permission to use the survey instrument. This study was part of the broader project titled *Development and Evaluation of Speaking Up Training Program*. One review study from this project has been published [33], while two studies, focused on qualitative metasynthesis and instrument validation, are currently under review. Each study was conducted with different subjects and for difference purposes.

### 2.6. Analysis

All statistical analyses were completed in Stata MP Version 18. Descriptive statistics were used to summarize the general characteristics of nurses and their working environment. Speaking-up-related behaviors, organizational culture, teamwork climate, and working conditions were reported as mean and standard deviations. The Kolmogorov–Smirnov test was used to confirm the normality of the data. An independent *t*-test and one-way analysis of variance (ANOVA) were used to examine differences in speaking-up-related behaviors based on participants' characteristics, with a post hoc analysis performed using Scheffe's method. Statistical significance was determined by a *p* value of 0.05.

A cluster analysis was conducted using a two-step approach to identify clusters of nurses by their speaking-up behavior. According to Okuyama, Nakagami-Yamaguchi, and Hayakawa [34], cluster analysis effectively identifies homogeneous subgroups of nurses and recognizes patterns within a complex clinical environment. In our study, nurses were categorized into subgroups based on their upregulated speaking-up-related behaviors (perceived concerns, withholding voices, and speaking up). The first step adopted hierarchical clustering methods with an agglomerative approach and Ward's linkage between clusters. Scores for three domains of speaking-up-related behavior (i.e., perceived concerns, withholding voice, and speaking up) were included in hierarchical clustering analyses. First, the *Z* score of the sum of individual domain scores was calculated to standardize continuous variables. To determine the number of clusters, a dendrogram for estimating the number of likely clusters within participants was used. In addition, the Calinski–Harabasz pseudo-F statistics and the Duda–Hart index were considered to determine the number of clusters [35]. After deciding the number of clusters, data were subjected to K-mean cluster analyses to determine the final cluster centers. The level of speaking-up-related behavior for each cluster was presented, and differences among clusters were analyzed using one-way ANOVA. Differences in general characteristics among clusters were examined through Chi-square tests and one-way ANOVA. In addition, analysis of covariance (ANCOVA) was conducted to analyze differences in nursing organizational culture, teamwork climate, and working conditions while controlling for significant variations in general characteristics among clusters.

## 3. Results

### 3.1. Nurses' Characteristics and Their Speaking-Up-Related Behaviors

Table 1 presents characteristics of 597 nurses participating in the survey and their speaking-up behaviors. Mean age of all participants was 35.49 years. Approximately 95% of the nurses were women. Approximately 60% of the nurses worked in hospitals located in large cities, including the capital and metropolitan areas. The distribution by the hospital type was similar, with approximately 30%–40% in tertiary hospitals, general hospitals, and hospitals. Nurses from various departments participated in this study. Approximately 50% of the nurses worked in the general care unit, including the general ward and integrated care unit. Those working in special units, such as the ICU, ER, and OR, accounted for 21.8%. The proportion of nurses with limited experience, defined as less than 3 years, was 14.4%. Most (78.9%) nurses did not receive a speaking-up education.

Speaking-up-related behaviors, including perceived concerns, withholding voice, and speaking up, were statistically different depending on nurses' characteristics. Perceived concerns showed statistical differences by the hospital type and working department. The frequency of perceived concerns in tertiary and general hospitals was higher than that in hospitals (*p* < 0.001). Among departments, the frequency of perceived concerns was highest in the general ward, statistically higher than in other parts (*p* < 0.001). Withholding voice and speaking up were statistically different depending on clinical experience. Although the frequency of perceived concerns was similar regardless of clinical experience, nurses with limited experience exhibited a higher frequency of withholding voice (*p* < 0.001) and a lower frequency of speaking up (*p* < 0.001) compared with the nurses with longer experience.

### 3.2. Clusters of Nurses by Speaking-Up-Related Behavior

**Figure 1:**
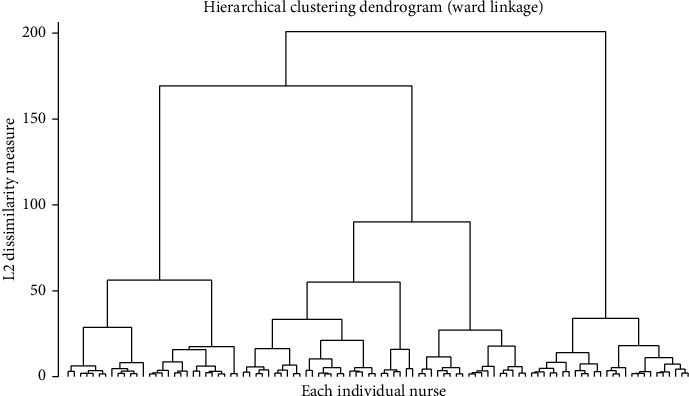
Hierarchical clustering dendrogram.

**Figure 2:**
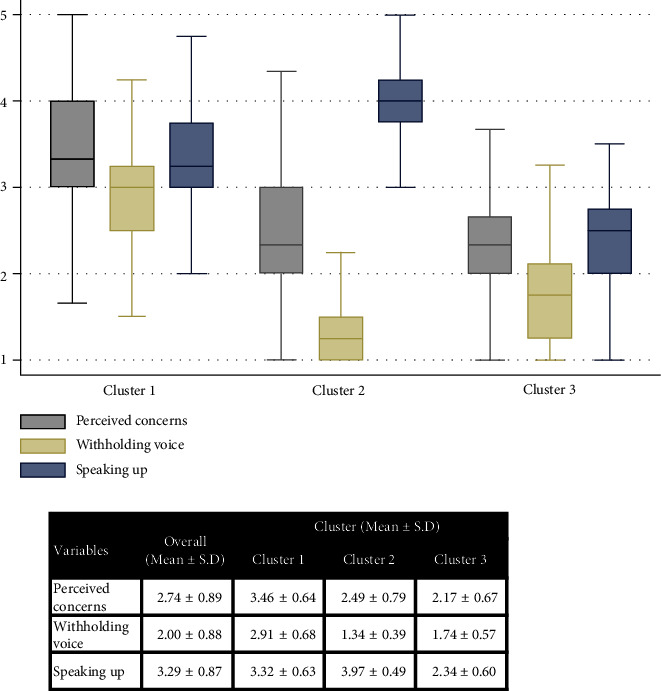
Level of perceived concerns, withholding voice, and speaking up by clusters. Note: the differences in all speaking-up-related behaviors among clusters were found to be statistically significant; perceived concerns (*F* = 175.61, *p* < 0.001), withholding voice (*F* = 443.83, *p* < 0.001), and speaking up (*F* = 392.65, *p* < 0.001).

The number of clusters was decided based on Dendrogram, simultaneously considering the Calinski–Harabasz pseudo-F statistics and Duda–Hart index to determine the number of clusters (Figure 1, Table 2). Three clusters were optimal according to the Dendrogram and Calinski–Harabasz pseudo-F statistics. Although it was anticipated that more than three clusters would be optimal according to the Duda–Hart index, this study ultimately decided on three clusters, considering the number of nurses in each cluster and the pattern of speaking-up-related behavior in each cluster simultaneously. Significant differences were observed in speaking-up-related behaviors by cluster: perceived concerns (*F* = 175.61, *p* < 0.001), withholding voice (*F* = 443.83, *p* < 0.001), and speaking up (*F* = 392.65, *p* < 0.001) (Figure 2). Thirty-five percent (*n* = 208) of the nurses were categorized into Cluster 1, comprising those with relatively high perceived concerns (3.46 ± 0.64), moderate withholding (2.91 ± 0.68), and moderate speaking up (3.32 ± 0.63) compared with other clusters. Thirty-seven percent (*n* = 221) of the nurses were categorized into Cluster 2, with moderated perceived concerns (2.49 ± 0.79), low withholding (1.34 ± 0.39), and high speaking up (3.97 ± 0.49). Twenty-eight percent (*n* = 168) of the nurses were categorized into Cluster 3, with moderate perceived concerns (2.17 ± 0.67), low withholding (1.74 ± 0.57), and low speaking up (2.24 ± 0.60).

### 3.3. Differences in General Characteristics, Organizational Culture, Team Climate, and Working Conditions Among Clusters

Distributions of all general characteristics except clinical experience showed no significant differences among the three clusters (Table 3). Regarding clinical experience, 44.2% of the nurses with limited experience of less than 3 years were in Cluster 1, the highest proportion, while nurses with more experience were predominantly in Cluster 2 (*p*=0.027). The results of the comparison of organizational culture, teamwork climate, and working conditions between the clusters are presented in Table 4. Since clinical experience differed significantly between clusters, differences in organizational culture, teamwork climate, and working conditions were analyzed while controlling for its influence. Collaborative (*p* < 0.001), creative (*p* < 0.001), and hierarchical (*p*=0.006) cultures among nursing organizational cultures differed statistically according to clusters. Collaborative culture and creative culture were the highest in Cluster 2, while hierarchical culture was the lowest in Cluster 1. The level of competitive culture did not differ significantly among clusters. In addition, teamwork climate and working conditions were statistically different according to clusters (*p* < 0.001). Both teamwork climate and working conditions were the highest in Cluster 2 but the lowest in Cluster 1.

## 4. Discussion

Nurses' speaking-up-related behaviors were categorized into three clusters. Cluster 1 exhibited high perceived concerns, moderate withholding, and moderate speaking up. Cluster 2 showed moderated perceived concerns, low withholding, and high speaking up. Cluster 3 had moderate perceived concerns, low withholding, and low speaking up. Cluster 2 is regarded as the most assertive group, demonstrating little withholding and very high speaking up despite moderate perceived concerns. Conversely, Cluster 3 is considered the most vulnerable group, with the lowest scores in both perceived concerns about patient safety and frequency of speaking up compared with all other clusters. Cluster 1 is characterized by the recognition of many concerns related to patient safety, yet the frequency of speaking up is low compared with the frequency of perceived concerns, potentially influenced by various factors. The findings presented in this study contribute novel insights to the existing literature on nurses' speaking-up-related behaviors. The SUPS-Q, which assesses nurses' speaking-up-related behaviors, has emerged relatively recently in the literature [29]. Previous studies have primarily examined individual outcomes of each speaking-up-related behavior. However, this study highlights the interconnectedness of perceived concerns, withholding voice, and speaking up, revealing correlations among these behaviors [5, 36]. Therefore, a comprehensive analysis of behavior patterns is essential for understanding nurses' speaking-up tendencies.

Among general characteristics, cluster distribution showed statistical differences depending on clinical experience. Nurses with limited experience had higher proportions of Cluster 1 and Cluster 3 than those with more experience, whereas the proportion of nurses in Cluster 2, characterized by proactive speaking-up behavior, was lower among nurses with limited experience than in those with more experience. By clinical experience, perceived concerns were not statistically different between nurses with limited experience and those with more experience. However, both withholding voice and speaking up were statistically different by clinical experience in this study. The low speaking-up competency of nurses with limited experience is aligned with previous studies showing that nurses with less experience are more hesitant to speak up than nurses with more experience [25, 37]. Hierarchical structures known to lead to a psychologically unsafe culture have been identified as significant barriers to speaking up among healthcare professionals [17, 38]. The hierarchical structure often discourages nurses from speaking up to higher-level nurses and physicians [14, 17]. Given that less experienced nurses typically occupy lower positions within the organizational hierarchy, they might be particularly susceptible to the negative impact of a hierarchical organizational structure. However, in our study, the hierarchical culture score was lower in Cluster 1 than in Cluster 2. This may be influenced by a lower sensitivity to hierarchical culture among less experienced nurses. Our findings align with previous studies conducted in Korean settings, which indicated that nurses with over 3 years of experience score higher in hierarchical culture [39]. Nurses in Cluster 1, who are generally younger and less experienced, may not fully recognize hierarchical dynamics and might accept them as a natural part of the organizational culture, particularly in Asian contexts where respect for age and seniority is strongly emphasized. It is essential to recognize that nurses' perceptions of hierarchical culture were predominant among this study's four types of cultures. This underscores its deep entrenchment in healthcare settings despite the negative impact of hierarchical culture on patient safety [14, 40]. To ensure patient safety, it is imperative to cultivate a culture that prioritizes patient safety over hierarchical structures, and improving nurses' sensitivity to hierarchical culture is essential for enhancing this organizational culture.

Although hierarchical culture scores were high across the organization, the variables with particularly high scores in groups with more active speaking up behaviors (Cluster 2) were teamwork climate, collaborative culture, and working conditions. Nurses in Cluster 2 had a more favorable perception of collaborative and creative organizational culture, teamwork climate, and working conditions. This finding aligns with previous cluster analyses of nurses, which showed that subgroups characterized by positive team relationships and hospital administrative support had a more positive attitude toward speaking up compared with other clusters characterized by lower team relationships [34]. Regarding organizational culture, a collaborative (clan) culture values collaboration, consensus, and the opinions of all employees. A creative (adhocracy) culture refers to a culture in which decision-making is characterized by decentralized decision-making, a flat hierarchy, and strong mutual support among team members [31]. Thus, both cultures emphasize teamwork, supporting significant relationship according to several studies [41, 42].

Effective teamwork has been revealed as an essential component of patient safety in healthcare settings where various professionals collaborate [43, 44]. It can promote collaboration and coordination among healthcare team members, ensuring safe care and swift emergency response [45, 46]. In addition, it facilitates accurate and timely communication about patient care [1, 43, 46] and enhances mutual support, enabling early risk identification and prevention of patient safety incidents [23, 47]. In this study, Cluster 2, the most assertive group, exhibited the highest level of teamwork climate, consistent with other studies [10, 22]. Several studies have indicated that speaking up or withholding voice behavior is influenced by the relationship with voice target [6, 14, 25] and the overall teamwork climate [22]. Given that these behaviors occur within interpersonal dynamics, the quality of relationships and the collaborative atmosphere within the team play significant roles.

Lastly, working conditions related to patient safety were statistically higher in Cluster 2 and Cluster 3 than in Cluster 1. Working conditions involving sufficient staffing, training systems, information accessibility, and adequate supervision influence teamwork and patient safety [48, 49]. In the present study, nurses' perceived concerns varied by the hospital type and department. Since institutional environments differ by severity and workload (e.g., tertiary and general hospitals often present higher severity and workload variations), these factors could impact nurses' speaking-up behaviors, aligning with previous research [49, 50]. In addition, it was noteworthy that Cluster 1, which had the lowest level of working conditions, exhibited a higher frequency of perceived concerns and withholding voice than Clusters 2 and 3. Poor working conditions for healthcare professionals cannot be a safe environment for patients. Thus, healthcare professionals in such organizations are more likely to be unable to provide safe care and encounter many situations that threaten patient safety. Moreover, poor work environments, such as heavy workload, resource constraints, and/or unclear guidelines, can hinder nurses' voice behaviors [16, 21, 51]. According to a qualitative study by Mawuena and Mannion [21], nurses with a high workload may choose to remain silent to avoid the overwhelming obstacle of additional work required to address threats to patient safety. Finding of the present study demonstrating a high level of withholding voice in the group with low working conditions supports the results of Mawuena and Mannion [21].

### 4.1. Strengths and Limitations

The strengths of our study include utilizing cluster analysis to classify subgroups of nurses based on their speaking-up-related behaviors and analyzing the differences among these subgroups, with a focus on their perceptions of organizational culture. This study has some limitations. First, the collection of speaking-up-related behaviors relied on self-reported questionnaires, which could introduce social desirability bias in nurses' responses. Second, caution should be exercised when generalizing findings to other professional groups as this study surveyed a single professional group. Furthermore, the internal consistency of the competitive culture among organizational cultures was low, indicating insufficient internal consistency in this measurement. Finally, although speaking-up behavior was used as a clustering variable based on previous literature, it is important to recognize that other factors, such as the significantly differing clinical nursing experience among the clusters in our sample, may also influence the results.

### 4.2. Implications for Nursing Management

Our findings on subgroups of nurses based on speaking-up-related behavior have significant implications for nursing management. We discovered that perceptions of organizational culture, teamwork climate, and working conditions varied among different nurse subgroups. This highlights the need for tailored education programs that address the specific characteristics of each group, rather than relying on uniform training approaches. Healthcare institutions should assess nurses' speaking-up-related behaviors and provide targeted training based on these assessments. Notably, the ideal subgroup identified in our study exhibited high teamwork, favorable working conditions, and collaborative and creative organizational culture. To enhance speaking up behaviors, nurse managers or hospitals should implement safe working environments and foster a safe culture that prioritizes patient safety. This organizational improvement will increase the speaking up behavior among nurses.

## 5. Conclusion

In conclusion, discussing patient safety concerns is essential for ensuring patient safety. Our results indicate that the ideal nurse subgroup operates within a nursing organizational culture that prioritizes teamwork, effective collaboration, and a safe working environment. These factors play a significant role in promoting proactive speaking behaviors among nurses, contributing to enhanced patient safety.

## Figures and Tables

**Table 1:** General characteristics and speaking-up-related behavior.

Variables	*n*	(%)	Perceived concerns			Withholding voice			Speaking up		
			Mean ± S.D		*t*/*f*(*p*)	Mean ± S.D		*t*/*f*(*p*)	Mean ± S.D		*t*/*f*(*p*)
Total	597	(100.0)	2.74	±0.89		2.00	±0.88		3.29	±0.87	
Gender											
Men	33	(5.5)	2.80	±1.07	0.41	2.27	±1.20	1.77	3.42	±0.64	−0.89
Women	564	(94.5)	2.73	±0.88	(0.681)	1.99	±0.86	(0.077)	3.28	±0.88	(0.372)
Age, M ± SD	35.49	±8.50									
Location											
Capital	149	(24.9)	2.76	±0.94	0.60	1.99	±0.90	1.31	3.24	±0.92	0.30
Metropolitan	210	(35.2)	2.78	±0.89	(0.548)	2.08	±0.89	(0.272)	3.30	±0.85	(0.741)
Nonmetropolitan	238	(39.9)	2.69	±0.87		1.94	±0.86		3.30	±0.85	
Hospital type											
Tertiary hospital^1^	180	(30.1)	2.83	±0.85	7.90	1.94	±0.84	1.98	3.26	±0.83	1.09
General hospital^2^	183	(30.7)	2.87	±0.90	(< 0.001)	2.11	±0.96	(0.138)	3.36	±0.84	(0.337)
Hospital^3^	234	(39.2)	2.56	±0.89	(1 > 3, 2 > 3)∗	1.96	±0.85		3.24	±0.92	
Department											
General ward^1^	159	(26.6)	2.93	±0.90	4.69	2.10	±0.94	1.85	3.29	±0.77	1.02
Integrated care unit^2^	130	(21.8)	2.75	±0.87	(< 0.001)	2.06	±0.87	(0.136)	3.19	±0.92	(0.383)
Special unit^3^	130	(21.8)	2.70	±0.90	(1 > 4)∗	1.98	±0.87		3.27	±0.86	
Others^4^	178	(29.8)	2.57	±0.86		1.88	±0.85		3.36	±0.91	
Clinical experience											
M ± SD	9.86	±7.24									
≤ 3 year	86	(14.4)	2.89	±0.84	−1.71	2.34	±0.89	−3.90	3.07	±0.79	2.54
> 3 year	511	(85.6)	2.71	±0.90	(0.088)	1.94	±0.87	(< 0.001)	3.32	±0.87	(0.011)
Speaking-up education											
Yes	126	(21.1)	2.74	±0.84	−0.03	1.98	±0.94	0.27	3.30	±0.87	−0.26
No	471	(78.9)	2.74	±0.90	(0.975)	2.01	±0.87	(0.789)	3.28	±0.87	(0.793)

*Note:* The special unit included the intensive care unit (ICU), emergency room (ER), and operating room (OR).

^∗^Post hoc analysis results reported only statistically significant findings.

^1–4^ *p* < 0.05.

**Table 2:** Index for determining the number of clusters.

Calinski–Harabasz		Duda–Hart		
#C	Pseudo-*F*	#C	Je(2)/Je(1)	Pseudo *T*-squared
		1	0.7801	167.77
2	167.77	2	0.7130	165.02
3	178.99	3	0.7304	73.82
4	144.56	4	0.8092	49.06
5	127.91	5	0.8729	26.64
6	115.21	6	0.8296	32.25
7	105.36	7	0.8570	23.20
8	98.88	8	0.8680	16.88
9	91.26	9	0.8297	19.91
10	84.77	10	0.8445	18.60
11	79.10	11	0.8032	12.00
12	73.88	12	0.8650	13.26
13	70.35	13	0.8395	14.34
14	67.20	14	0.8753	11.40
15	64.31	15	0.8745	11.48

**Table 3:** Comparison of general characteristics among three clusters.

Variables	*n*	Cluster 1 *n* (%)		Cluster 2 *n* (%)		Cluster 3 *n* (%)		Chi2/*F*	*p* value
Total	597	208	(34.9)	221	(37.0)	168	(28.1)		
Gender									
Men	33	14	(42.4)	12	(36.4)	7	(21.2)	1.18	0.555
Women	564	194	(34.4)	209	(37.1)	161	(28.5)		
Location									
Capital	149	51	(34.2)	53	(35.6)	45	(30.2)	1.71	0.789
Metropolitan	210	77	(36.7)	73	(34.8)	60	(28.5)		
Nonmetropolitan	238	80	(33.6)	95	(39.9)	63	(26.5)		
Hospital type									
Tertiary hospital	180	61	(33.9)	71	(39.4)	48	(26.7)	4.31	0.365
General hospital	183	73	(39.9)	64	(35.0)	46	(25.1)		
Hospital	234	74	(31.6)	86	(36.8)	74	(31.6)		
Department									
General ward	159	62	(39.0)	58	(36.5)	39	(24.5)	8.71	0.191
Integrated care unit	130	52	(40.0)	39	(30.0)	39	(30.0)		
Special unit	130	43	(33.1)	47	(36.1)	40	(30.8)		
Others	178	51	(28.7)	77	(43.2)	50	(28.1)		
Clinical experience									
M ± SD (years)		7.83	±5.45	11.91	±7.99	9.69	±7.44	18.03	<0.001
≤ 3 years	86	38	(44.2)	21	(24.4)	27	(31.4)	7.21	0.027
> 3 years	511	170	(33.3)	200	(39.1)	141	(27.6)		
Education									
Yes	126	44	(34.9)	49	(38.9)	33	(26.2)	0.37	0.832
No	471	164	(34.8)	172	(36.5)	135	(28.7)		

**Table 4:** Comparison of organizational culture, teamwork climate, and working conditions between three clusters.

Variables	Total		Cluster 1 (*n* = 208)		Cluster 2 (*n* = 221)		Cluster 3 (*n* = 168)		*F*	*p* value	Post hoc
	(Mean ± S.D)		(Mean ± S.D)		(Mean ± S.D)		(Mean ± S.D)				
Organizational culture											
Collaborative	3.10	±0.86	2.78	±0.81	3.40	±0.84	3.09	±0.81	29.18	< 0.001	2 > 3 > 1
Creative	2.89	±0.72	2.67	±0.64	3.14	±0.75	2.83	±0.69	24.21	< 0.001	2 > 1.3
Hierarchy	3.48	±0.66	3.36	±0.76	3.53	±0.59	3.56	±0.58	5.13	0.006	2.3 > 1
Competitive	2.86	±0.61	2.84	±0.61	2.92	±0.61	2.82	±0.62	1.29	0.276	
Teamwork climate	3.21	±0.76	2.85	±0.72	3.51	±0.72	3.25	±0.69	42.57	< 0.001	2 > 3 > 1
Working conditions	2.93	±0.77	2.68	±0.74	3.13	±0.75	2.97	±0.76	116.71	< 0.001	2.3 > 1

*Note:* Differences in organizational culture, teamwork, and working conditions across clusters were analyzed using ANCOVA, with clinical experience as a covariate due to its significant variation among clusters. The post-hoc analysis results reported only statistically significant findings.

## Data Availability

The data that support the findings of this study are available from the corresponding author upon reasonable request.
